# Mesenchymal stem cell-derived extracellular vesicles for immunomodulation and regeneration: a next generation therapeutic tool?

**DOI:** 10.1038/s41419-022-05034-x

**Published:** 2022-07-04

**Authors:** Meng Kou, Li Huang, Jinjuan Yang, Zhixin Chiang, Shaoxiang Chen, Jie Liu, Liyan Guo, Xiaoxian Zhang, Xiaoya Zhou, Xiang Xu, Xiaomei Yan, Yan Wang, Jinqiu Zhang, Aimin Xu, Hung-fat Tse, Qizhou Lian

**Affiliations:** 1 Cord Blood Bank Centre, Guangzhou Women and Children’s Medical Centre, Guangzhou Medical University, Guangzhou, China; 2 Department of Allied Health Sciences Faculty of Science, Tunku Abdul Rahman University, Ipoh, Malaysia; 3 State Key Laboratory of Pharmaceutical Biotechnology, the University of Hong Kong, Hong Kong SAR, China; 4 Department of Medicine, the University of Hong Kong, Hong Kong SAR, China; 5 Department of Stem Cell & Regenerative Medicine, State Key Laboratory of Trauma, Burn and Combined Injury, Daping Hospital, Army Medical University, Chongqing, 400042 China; 6 Department of Chemical Biology, The MOE Key Laboratory of Spectrochemical Analysis & Instrumentation, College of Chemistry and Chemical Engineering, Xiamen University, Xiamen, Fujian 361005 China; 7 Xiamen Cardiovascular Hospital of Xiamen University, School of Medicine, Xiamen University, Xiamen, China; 8 HKUMed Laboratory of Cellular Therapeutics, the University of Hong Kong, Hong Kong SAR, China; 9 Department of Surgery, Shenzhen Hong Kong University Hospital, Shenzhen, 518053 China

**Keywords:** Mesenchymal stem cells, Regeneration

## Abstract

Mesenchymal stem cells (MSCs) can be widely isolated from various tissues including bone marrow, umbilical cord, and adipose tissue, with the potential for self-renewal and multipotent differentiation. There is compelling evidence that the therapeutic effect of MSCs mainly depends on their paracrine action. Extracellular vesicles (EVs) are fundamental paracrine effectors of MSCs and play a crucial role in intercellular communication, existing in various body fluids and cell supernatants. Since MSC-derived EVs retain the function of protocells and have lower immunogenicity, they have a wide range of prospective therapeutic applications with advantages over cell therapy. We describe some characteristics of MSC-EVs, and discuss their role in immune regulation and regeneration, with emphasis on the molecular mechanism and application of MSC-EVs in the treatment of fibrosis and support tissue repair. We also highlight current challenges in the clinical application of MSC-EVs and potential ways to overcome the problem of quality heterogeneity.

## Facts

- MSC-derived EVs have low-immunogenicity and strong potential for therapeutic applications.
- MSC-derived EVs were used to treat tissue fibrosis and promote tissue regeneration.
- MSC-derived EVs are proposed as a novel therapeutic agent to mediate immunomodulation and promote regeneration.

## Open questions

- How can MSC-derived EVs mediate immunomodulation and regeneration?
- How can MSC-derived EVs be used to aid regeneration of fibrotic tissue?
- How can mass manufacturing of MSC-derived EVs be achieved and the problem of quality heterogeneity overcome?
- What are the challenges of MSC-derived EV-based immunomodulation and regeneration in clinical practice?

## Introduction

Mesenchymal stem cells (MSCs) exist in various tissues such as bone marrow (BMSCs), umbilical cord blood (UC-MSCs) and umbilical cord tissue, placental tissue (hPMSCs), adipose tissue (ADSCs), and menstrual blood (MenSCs). These cells have multidirectional differentiation potential [1] to become osteoblasts, chondrocytes or adipocytes in vitro [2], and have a unique function of cytokine secretion [3]. Cell models have been applied in proliferation, transplantation, and differentiation studies, and in identification of immune responses in vitro [4]. Numerous studies have shown that MSCs have great potential in immune regulation and regeneration [5]. The U.S. FDA has approved nearly 60 clinical trials [6], mainly focused on Hematopoietic Stem Cell Transplantation (HSCT) [7], tissue healing, Autoimmune Disease (AID), and genetic therapy vectors [8]. Recently, MSCs have been widely used in clinical studies as a regenerative agent and to treat a variety of conditions including osteoarthritis [9], pulmonary fibrosis, spinal cord injury, myocardial damage, knee cartilage injury, dental pulp regeneration, and organ transplantation [10]. An increasing number of studies has revealed that the powerful therapeutic effects of MSCs are due to paracrine-like secretion of cytokines (growth factors and chemokines) [11, 12] and extracellular vesicles (EVs) as well as their involvement in cellular communication [13–16].

Application of MSCs as cell therapy is based on regulating the inflammatory response and participating in tissue repair and regeneration [17]. The therapeutic effect of MSCs is mainly attributed to their immunomodulatory function regulated by the inflammatory environment [18]. When stimulated by inflammatory factors, MSCs produce a large number of immunomodulatory factors, cell chemokines, and growth factors, thereby regulating the tissue immune microenvironment and promoting tissue regeneration [19]. There is accumulating evidence that EVs derived from MSCs preserve the therapeutic action of the parent MSCs and their use avoids the safety concerns associated with live cell therapy [20, 21]. Therefore, use of MSC-EVs to replace MSCs as cell-free therapy may be the focus of future clinical treatments [20]. We review recent studies of the role of MSC-EVs in immunomodulation and regeneration, focusing on their molecular mechanisms in the treatment of osteoarthritis, spinal cord injury, skin injury, and liver, kidney, and lung fibrosis.

## Extracellular vesicles composition

**Fig. 1:**
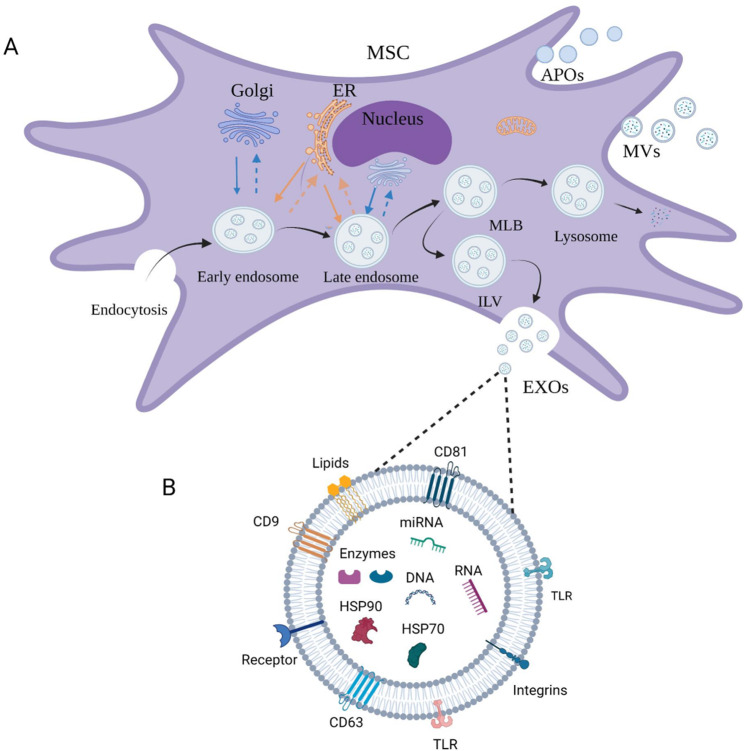
The development and main types of extracellular vesicles. **A** Exosomes are derived from the endosomal pathway. **B** Composition of exosomes.

Extracellular vesicles (EVs) exist in body fluids, are released by cells, and have a membrane structure [22]. They can be divided into four subgroups according to their diameter: exosomes (30–150 nm), microvesicles (100–1000 nm), apoptotic bodies (50–5000 nm, generated during cell apoptosis) [23, 24], and oncosomes (1–10 μm), newly discovered and observed in cancer cells [25]. EVs encapsulate many bioactive molecules (proteins, lipids, nucleic acids, and organelles) [26–28] that can be delivered to target cells. Large amounts of data suggest that exosomes and microvesicles are vital mediators of EVs in numerous physiological (pathological) processes [29] (Fig. 1).

### Exosomes

Exosomes are microscopic vesicles with a density of 1.11–1.19 g/mL. They have a typical “disk-like” structure and flat spherical shape when seen under an electron microscope [24]. Many kinds of cellsin various body fluids and cell supernatants can secrete exosomes under normal and pathological conditions. Exosomes were first discovered in 1983 in sheep reticulocytes and were named “Exosomes” by Johnstone in 1987 [30]. These tiny vesicles contain specific proteins, lipids, and nucleic acids that can be transmitted and serve as signaling molecules to alter the function of other cells [31, 32].

During the formation of exosomes, the extracellular components and cell membrane proteins are wrapped by the invaginated plasma membrane to form early endosomes. These can exchange materials with intracellular organelles and develop into late endosomes, eventually forming intracellular multivesicular bodies (MVBs) [33, 34]. MVBs contain many intraluminal vesicles (ILVs) [35]. They may be degraded and released into the cytoplasm by fusion with autophagosomes or lysosomes, or released into extracellular vesicles by fusion with plasma membrane, including ILVs, resulting in exosome formation [34]. Exosome-mediated intercellular communication is achieved by direct membrane fusion, receptor-mediated endocytosis, phagocytosis, caveolae, and micropinocytosis [36–38].

Proteins involved in exosome biogenesis (such as transport and fusion) include Rab GTPases [39–41], ESCRT (endosomal sorting complex required for transport) [42], annexin, lipid raft proteins, and four transmembrane proteins (CD63, CD81, and CD9) [43, 44]. In addition, they also contain biosynthetic antibodies (Alix and TSG101) involved in MVBs [45, 46], cholesterol, ceramide, phosphoglyceride that provides structural stability, and immune-related molecule MHC-II that is involved in antigen binding and presentation. Exosomes also carry functional mRNAs and miRNAs that can be transferred between cells [47]. Exosomes released by tumors contain single-stranded DNA, genomic DNA, cDNA, and a transposable element [48, 49]. It is clear that exosomes have many functions as biomarkers of disease.

### Microvesicles

Microvesicles are also known as microparticles. Biogenesis of MVs differs to that of exosomes since they are released from outward budding and fission of plasma membrane when the cell is stimulated or apoptotic [50]. Nonetheless, they share characteristics of high biocompatibility, and low immunogenicity and targeting and can be used as drug carriers [51]. Studies have shown that the use of tumor cell-derived MVs to deliver chemotherapy drugs produces in better cancer treatment results with few side effects or adverse reactions [52, 53].

## MSC-derived extracellular vesicles

Although MSCs derive from a variety of sources, they can all be adherent in culture and differentiated into a variety of cell types with specific surface markers [54]. With the need for clinical treatment with MSCs, the Mesenchymal and Tissue Stem Cell Committee of the International Society for Cellular Therapy (ISCT) has proposed minimum criteria for identification of human MSCs: (1) Cultured under standard conditions they must adhere to plastic substrates; (2) On flow cytometry, the positive rate of CD105, CD73 and CD90 expression in MSC surface markers should reach 95%, and negative expression rate CD45, CD34, CD14 or CD11b, CD79a or CD19 or HLA-DR (human leukocyte antigen -DR) (≤2% positive); (3) After induction by standard methods in vitro, MSCs must be able to induce differentiation into osteoblasts, chondrocytes and adipocytes [55]. Nonetheless, further research has revealed that these standards do not fully define MSCs [56]. There is accumulating evidence that heterogeneous MSCs have multiple cell subpopulations with characteristic surface markers [57, 58], but the definition of surface markers and biological functions of these subpopulations requires ongoing exploration.

MSCs are easy to resuscitate and proliferate in vitro, enabling them to be mass-produced for clinical application [18]. In recent years, they have been the most studied stem cell type for clinical application, and have played an effective therapeutic role in graft-versus-host disease (GVHD) [7], kidney injury [59], tissue and organ transplantation, immune tolerance [60], nerve injury, rheumatic disease, and liver disease. At present, MSCs have attracted much attention in the context of the COVID-19 pandemic [61]. Leng et al. demonstrated that in an MSC treatment group, patients with COVID-19 infection were cured or their condition significantly improved as a result of regulation of increased interleukin 10 (IL 10) expression, inhibition of overactivated immune T cells and NK cells, and a significantly reduced TNF-α level [62].

Despite their advantages, there are aspects of MSC therapy that warrant consideration. First, the proliferation ability of MSCs is gradually weakened and accompanied by a certain degree of differentiation and even aging with increasing passages during in vitro culture. This impacts their regulatory and therapeutic ability [56, 63]. Second, in the in vivo environment, heredity factors and the self-renewal ability of MSCs cannot be controlled with consequent potential for tumorigenicity [64]. In addition, although MSCs have a strong regenerative regulatory potential, it is uncertain whether they can target or remain at the damaged site following intravenous injection [65]. There is some evidence that only a small number of MSCs reach the target site due to the host body’s scavenging capacity [66, 67]. Although in-situ injection can partially solve these problems, there remain problems with cell differentiation and aging, and the clinical effects are not optimistic [68]. MSCs have also been found to cause and promote the growth of various types of cancer [69]. In addition, there are the usual associated risks of cell therapy such as viral infection and immune rejection as well as problems with storage and transportation [70].

The discovery that most therapeutic effects of MSCs depend on their paracrine action and that EVs can replace their parent cells offers exciting prospects for researchers [21]. EVs offer great advantages [71]: they are not self-replicating and largely avoid the risk of tumorigenicity [72]; compared with cell therapy, EVs are safer; as nanoparticles they have both biocompatibility and low immunogenicity, enabling them to cross-protective barriers such as the blood-brain barrier [73]; they can be continuously secreted by immortalized cells to obtain a sufficient number [74]; EVs protect their internal biomolecular activity via their lipid membrane structure, can be preserved for a prolonged period at -80°C, and are not subject to deactivation, even after repeated freezing and thawing [75, 76]; and they have an encapsulation capability, can load specific drugs and transport them to target cells [77].

Notably, MSC-EVs express EV surface markers CD63, CD9 and CD81, as well as mesenchymal stem cell surface markers CD44, CD73, and CD90 [78]. In addition, proteins contained in the extracellular vesicles secreted by MSCs are a specific protein subclass that determines their unique biological functions [36]. At the same time, the encapsulated mRNA and miRNA in MSC-EVs form the molecular basis for their function [79]. Accordingly, MSC-EVs transmit information and communicate with target cells through internal substances, thus changing the activity and function of target cells [80].

With their unique advantages, MSC-EVs play an important role in immune regulation and regeneration. Studies of the promotion of regeneration through immune regulation are described in detail below. Meanwhile, in the treatment of autoimmune diseases, Wu et al. found that BM-MSC-derived EVs targeted inhibition of the cyclin I-activated ATM/ATR/p53 signaling pathway by upregulation of miR- 34a, thereby inhibiting RA fibroblast-like synoviocytes (RA-FLSs) and significantly ameliorating RA inflammation in vivo [81]. Another study on the regulation of type-I autoimmune diabetes mellitus (T1DM) showed that AD-MSC-derived exosomes ameliorated T1DM symptoms by upregulating the expression of regulatory T cells, interleukin 4 (IL 4), IL 10 and transforming growth factor-beta (TGF-β) and down-regulating IL 17 and interferon-gamma (IFN-γ) [82]. Additional studies of autoimmune disease regulation have been summarized elsewhere [83]. Recently MSC-EVs have also been applied in clinical practice. Nassar et al. are in the process of evaluating the effect of human UC-MSC-derived EVs on islet β cells in patients with T1DM (trial NCT02138331). Recent clinical trials have been conducted to evaluate the safety and efficacy of MSC-EVs in patients with a variety of diseases based on their potential for immune regulation and regeneration (Table 1).

**Table 1:** Summary of registered clinical trials based on MSC-EVs with potential for immune regulation and regeneration.

Register No.	Title	Phase	Condition	Intervention	URL
NCT05127122	Bone Marrow Mesenchymal Stem Cell-Derived Extracellular Vesicles Infusion Treatment for ARDS	I/II	ARDS	BMSC-EVs; IV	https://ClinicalTrials.gov/show/NCT05127122
NCT04493242	Extracellular Vesicle Infusion Treatment for COVID-19 Associated ARDS	II	COVID-19 ARDS	BMSC-EVs; IV	https://ClinicalTrials.gov/show/NCT04493242
NCT05078385	Safety of Mesenchymal Stem Cell Extracellular Vesicles (BMSC-EVs) for the Treatment of Burn Wounds	I	Burn wounds	BMSC-EVs; apply to wound	https://ClinicalTrials.gov/show/NCT05078385
NCT05130983	A Phase I Study of ExoFlo, an ex Vivo Culture-expanded Adult Allogeneic Bone Marrow Mesenchymal Stem Cell-Derived Extracellular Vesicle Isolate Product, for the Treatment of Medically Refractory Crohn’s Disease	I	Crohn’s Disease	BMSC-EVs; IV	https://ClinicalTrials.gov/show/NCT05130983
NCT04657458	Expanded Access Protocol on Bone Marrow Mesenchymal Stem Cell-Derived Extracellular Vesicle Infusion Treatment for Patients With COVID-19 Associated ARDS	open-label	Critically ill COVID-19 ARDS	BMSC-EVs; IV	https://ClinicalTrials.gov/show/NCT04657458
NCT05125562	Bone Marrow Mesenchymal Stem Cell-Derived Extracellular Vesicles Infusion Treatment for Mild-to-Moderate COVID-19: A Phase II Clinical Trial	II	Mild-to-Moderate COVID-19	BMSC- EVs; IV	https://ClinicalTrials.gov/show/NCT05125562
NCT04327635	Safety Evaluation of Intracoronary Infusion of Extracellular Vesicles in Patients With AMI	I	AMI	EVs; Intracoronary infusion	https://ClinicalTrials.gov/show/NCT04327635
NCT05116761	ExoFlo™ Infusion for Post-Acute COVID-19 and Chronic Post-COVID-19 Syndrome	I/II	COVID-19	BMSC-EVs; IV	https://ClinicalTrials.gov/show/NCT05116761
NCT05176366	Study of ExoFlo for the Treatment of Medically Refractory Ulcerative Colitis	I	Ulcerative Colitis	BMSC-EVs; IV	https://ClinicalTrials.gov/show/NCT05176366
NCT04173650	MSC EVs in Dystrophic Epidermolysis Bullosa	I/II	DEB	BMSC-EVs; apply to wound	https://ClinicalTrials.gov/show/NCT04173650
NCT05215288	Intermediate Size Expanded Access for the Use of ExoFlo in the Treatment of Abdominal Solid Organ Transplant Patients Who Are at Risk of Worsening Allograft Function With Conventional Immunosuppressive Therapy Alone	I	Solid Organ Transplant Rejection	BMSC-EVs; IV	https://ClinicalTrials.gov/show/NCT05215288
NCT04223622	Effects of ASC Secretome on Human Osteochondral Explants	open-label	OA	ASC secretome;^*^ IV	https://ClinicalTrials.gov/show/NCT04223622
NCT04270006	Evaluation of Adipose-Derived Stem Cells Exo. in Treatment of Periodontitis	I	Periodontitis	ASC-EVs	https://ClinicalTrials.gov/show/NCT04270006

*AMI* acute myocardial infarction, *ARDS* acute respiratory distress syndrome, *ASC* adipose-derived stem cell, *BMSC* bone mesenchymal stem cell, *COVID-19* corona virus disease 2019, *DEB* dystrophic epidermolysis bullosa, *EVs* extracellular vesicles, *IV* intravenous administration, *OA* osteoarthritis.

^*^ASC secretome, either complete conditioned medium or EVs.

## Application of MSC-EVs in immune regulation and regeneration

The therapeutic potential of MSC-EVs has been reported in immune regulation and tissue regeneration based on EV-mediated cellular communication between MSCs and several target cells, including macrophages, microglia, chondrocytes, articular chondrocytes, endothelial cells, fibroblasts, pericytes, neural stem cells (NSC), neurons, hepatic stellate cells, and podocytes. In this paper, we discuss the molecular mechanisms of MSC-EVs in tissue repair and anti-fibrosis, in which several clusters of miRNA and their downstream pathways have been revealed to play important roles in osteoarthritis, spinal cord injury, skin injury, liver fibrosis, kidney fibrosis, and lung fibrosis (Tables 2–7).

**Table 2:** Summary of studies on the role of extracellular vesicles in osteoarthritis.

EVs source	Target cells or tissues	Animal model	Molecular mechanism	Action effect	Ref
BMSC-EVs	Chondrocytes	–	Downregulate TNF-α-induced expression of COX2, ILs and collagenase activity	Promote the production of proteoglycan, type II collagen, and chondrocytes regeneration	[85]
hBMSC-EVs	Chondrocyte	–	Downregulate IL-1ß-activated pro-inflammatory Erk1/2, PI3K/Akt, p38, TAK1, and NF-κB signaling pathways	Promote cell proliferation and migration and reduce apoptosis.	[86]
Murine BMSCs-EVs	OA-like chondrocytes	CIOA	Inhibit MMP-13, ADAMTS5 and iNOS	Reinduce the expression of type II collagen, aggrecan, and protected mice from joint damage	[87]
hBMSC-EVs	OA-like chondrocytes	OA	TGFBI inhibit cartilage and bone degradation, and limit calcification and osteophyte formation	Increase chondrocyte proliferation	[88]
BMSC-Exos	Macrophages	OA	Promote the conversion of RAW264.7 from M1 to M2, reduce the expression of IL-1β, TNF-α and IL-6, and enhance IL-10, chondrogenic genes, collagen II and sox9	Inhibit OA progression	[89]
hASC-EVs	Chondrocytes	MIA, DMM	Increase type collagen synthesis and decrease MMP-1, MMP-3, MMP-13, and ADAMTS-5 expression in the presence of IL-1β	Promote the proliferation and migration of human OA chondrocytes, and protected cartilage from degeneration	[90]
SMSC-EVs	Articular chondrocytes	OA	Highly-express miR-140-5p blocked ECM secretion decrease via RalA	Enhance proliferation, migration of chondrocytes, and prevent OA	[91]
SMSC-Exos	Articular chondrocytes	OA	Highly-expressed miR-155-5p promoted ECM secretion via Runx2	Enhance proliferation, migration of chondrocytes, and prevent OA	[92]
SMSC-EVs	Knee OA	Human knee OA patients	Encapsulate miR-31 ameliorates knee OA via the KDM2A/E2F1/PTTG1 axis.	Alleviate cartilage damage and inflammation in knee joints	[93]
BMSC-EVs	Chondrocyte	OA	Hypoxia increased the expression of miR-216a-3p and promoted down-regulation of JAK2	Promote proliferation, migration and reduce apoptosis	[94]
infrapatellar fat pad MSCs-Exos	Chondrocyte	OA	MiR100-5p-regulate inhibition of mTOR-autophagy pathway	Protect articular cartilage from damage and ameliorate gait abnormality in OA mice by maintaining cartilage homeostasis	[95]
UMSC-Exos	Chondrocyte	OA	Exosomal H19 against miR-29b-3p to upregulate FoxO3	Promote chondrocyte migration, matrix secretion, apoptosis suppression, as well as senescence suppression	[96]

*BMSC* bone mesenchymal stem cell, *CIOA* collagenase-induced osteoarthritis, *DMM* destabilization of the medial meniscus, *ECM* extracellular matrix, *EVs* extracellular vesicles, *Exos* exosomes, *hASC* human adipose-derived stem cell, *MIA* monosodium iodoacetate (induced osteoarthritis), *OA* osteoarthritis, *OA-CH* osteoarthritis-chondrocyte, *SMSC* synovial mesenchymal stem cell, *UMSC* umbilical cord mesenchymal stem cell.

**Table 3:** Summary of studies on the role of extracellular vesicles in spinal cord injury.

EVs source	Target cells or tissues	Animal model	Molecular mechanism	Action effect	Ref
hBMSC-Exos	Endothelial	SCI	Inhibit Bax and TNFα and IL 1β, and Bcl 2, IL 10 and angiogenesis	Attenuate the lesion size and improved functional recovery after SCI	[98]
BMSC-EVs	Pericyte	SCI	Inhibit NF-KB P65 signaling pathway	Ameliorate blood-spinal cord barrier	[99]
BMSC-Exos	Pericyte	SCI	Suppress the expression of caspase 1 and IL 1β by reducing pyroptosis	Ameliorate the motor ability of spinal cord injury rats	[100]
BMSC-EVs	NSCs	SCI	TGF-β enhanced the expression of Smad6	Promote the regeneration of neurons	[101]
BMSC-EVs	M2 macrophage	SCI	Up-regulate TGF-β, TGF-β receptor and relative proteins of tight junction	Improve locomotor recovery	[102]
hPMSC-Exos	Endogenous neural stem/progenitor cells	SCI	Promote NSCs proliferation and upregulate MEK, ERK, and CREB phosphorylation levels	Promote spinal cord functional recovery	[103]
MSC-EVs	DRG cells	SCI	Overexpress miR-381 up-regulates RhoA/ RHO kinase activity and down-regulate BRD4 expression and DRG cell apoptosis by inhibiting the BRD4/WNT5A axis	Promote SCI repair	[104]
MSC-Exos	Neurons	SCI	MiR-133b target down-regulates the expression of RhoA, and promotes ERK1/2 STAT3 and CREB signaling pathway	Improve the recovery of hindlimb locomotor function following SCI	[105]
BMSC-Exos	Neurons	MCAO	MiR-17-92 induces activation of mTOR/PI3K/Akt signaling pathway cascade	Enhance neuro-functional recovery of stroke	[106]
BMSC-Exos	Neurons	SCI	MiR-26a induces activation of PTEN/ Akt /mTOR signaling pathway cascade	Promote axonal regeneration and neurogenesis and attenuate glia scarring in SCI	[107]
BMSC-Exos	Microglia	SCI	Hypoxic exosomal miR-216a-5p modulate microglial polarization by TLR4/NF-κB/PI3K/AKT signaling cascades	Promote functional behavioral recovery after SCI	[108]
BMSC-EVs	Microglia	SCI	MiRNA-22 downregulates the expression of inflammatory cytokines and GSDMD	Nerve function repair after SCI	[109]
hUC-MSC-Exos	Neurons	SCI	MiR-199a-3p /145-5p affected TrkA ubiquitination and promoted the NGF/TrkA signaling pathway	Promote locomotor function in SCI rats	[110]

*BMSC* bone mesenchymal stem cell, *DRG* dorsal root ganglia *EVs* extracellular vesicles, *Exos* exosomes, *hPMSC* human placental mesenchymal stem cell, *MCAO* middle cerebral artery occlusion, *MSC* mesenchymal stem cell, *NSCs* neural stem cells, *SCI* spinal cord injury, *UC-MSC* umbilical cord mesenchymal stem cell.

**Table 4:** Summary of studies on the role of extracellular vesicles in skin injury.

EVs source	Target cells or tissues	Animal model	Molecular mechanism	Action effect	Ref
hADSC-Exos	–	Full-thickness skin defect model	Down-regulate TNF-α, IL-6, CD14, CD19, CD68, and C-caspase 3, up-regulate VEGF, CD31, Ki67, PCNA, filaggrin, loricrin and AQP3	Accelerate skin wound healing	[114]
hBMSC-Exos	HaCaT cells and HSFs	Full-thickness skin wounds injury model in rats	Target on TGF-β/Smad signaling pathway, but increased the expression of TGF-β3 and Smad7	Improve scar formation and promote wound healing	[115]
FDMSC-Exos	ADFs	Full-thickness dermal wound injury model	Inhibit MMP-13, ADAMTS5 and iNOS	Reinduce the expression of type II collagen, aggrecan, and protected mice from joint damage	[116]
hBMSC-Exos and JMMSC-Exos	Macrophages	Skin Wound-Healing	By carrying miR-223 targeting Pknox1	Induced macrophages toward M2 polarization and promote wound healing	[117]
mag-BMSC-Exos	HUVECs and HSFs	Rat Skin Wound Model	Highly-express miR-21-5p and target SPRY2 to activating PI3K/AKT and ERK1/2 signaling pathways	Accelerate skin wound healing	[118]
hUCMSCs-EVs	HaCaT cells and HSFs	Cutaneous wound mouse model	Highly-express miR-27b p and promote the expression of JUNB and IRE1α by targeting the Itchy E3 ubiquitin-protein ligase (ITCH)	Accelerate cutaneous wound healing	[119]
hUCMSC-Exos	Myofibroblast	Full-thickness skin defect mouse model	Highly-express microRNAs (miR-21, -23A, -125b and -145) repressed the TGF-β2 /SMAD2 pathway	Attenuate excess myofibroblast formation and anti-scarring	[120]
hADSC-Exos	HaCaT cells and HSFs	Wound healing of skin-injured mice	Highly-express miR-19b regulated TGF-β pathway by targeting CCL1	Promote the healing of skin wounds	[121]
hADSC-Exos	HSFs	Full-thickness skin defects in the backs of rats	Down-regulate the expression of Col1, Col3, α-SMA, IL-17RA, and P-SMad2 / P-SMad3, and up-regulate the level of SIP1, while overexpression miR-192-5p target inhibition of IL-17RA expression	Reduce the level of pro-fibrosis protein, improve hypertrophic scar fibrosis and accelerate wound healing	[122]
hADSC-EVs	HSFs and HMECs	–	Overexpression miR-486-5p inhibit Sp5 and elevate the CCND2 expression	Promote proliferation, migration and reduce apoptosis	[123]
hAMSC-Exos	Fibroblasts	Full-thickness skin defects in the backs of rats	Downregulation of LATS2 after overexpression of miR-135a	Increase cell migration and promote wound healing	[124]

*EVs* extracellular vesicles, *Exos* exosomes, *FDMSC* fetal dermal mesenchymal stem cell, *hADSC* human adipose-derived stem cell, *hAMSC* human amnion mesenchymal stem cell, *hBMSC* human bone mesenchymal stem cell, *HMEC* human microvascular endothelial cell, *HSF* Human skin fibroblast, *hUCMSC* human umbilical cord mesenchymal stem cell, *JMMSC* jaw bone marrow MSC.

**Table 5:** Summary of studies on the role of extracellular vesicles in liver fibrosis.

EVs source	Target cells or tissues	Animal model	Molecular mechanism	Action effect	Ref
hBMSC-Exos	Hepatic stellate cells	CCl4-induced liver fibrosis	Inhibited the expression of Wnt/β-catenin pathway, α-SMA, and Collagen I	Effectively alleviate liver fibrosis, and enhance liver functionality, hepatocyte regeneration	[126]
AMSC-EVs	Hepatic stellate cells	NASH, liver fibrosis	Decrease the number of KCs and the mRNA expression levels of TNF-α, IL1-β, IL 6, TGF-β, LPS, and TLR4	Improve liver inflammation and fibrosis	[127]
ADSC-Exos	HST-T6 cells^*^	Induced liver injury by CCl4	Down-regulate STAT3 and Bcl-2 and activated autophagy	Effective anti-liver fibrotic and attenuate liver injury	[128]
AMSC-Exos	Hepatic stellate cells	CCl4-induced liver fibrosis	miR-122	Enhance the therapeutic efficacy of AMSCs in the treatment of liver fibrosis	[129]
hTMSC-EVs	Human primary hepatic stellate cells	CCl4-induced liver fibrosis	MiR-486 inactivates hedgehog signaling	Attenuate HSC activation and liver fibrosis	[130]

*ADSC* adipose-derived mesenchymal stem cell, *AMSC* amnion-derived mesenchymal stem cell, *BMSC* bone mesenchymal stem cell, *CCl4* carbon tetrachloride, *EVs* extracellular vesicles, Exos exosomes, *HSC* hepatic stellate cell, *NASH* nonalcoholic steatohepatitis, *TMSC* tonsil-derived mesenchymal stem cell.

*HST-T6, mouse hepatic stellate cell line.

**Table 6:** Summary of studies on the role of extracellular vesicles in kidney fibrosis.

EVs source	Target cells or tissues	Animal model	Molecular mechanism	Action effect	Ref.
hUC-MSC-Exos	Kidney tissue	UUO	Through CK1δ/β-TRCP inhibited YAP activity	Ameliorate renal fibrosis	[132]
hUC-MSC-Exos	Renal tubular epithelial cells	UUO	Inhibit ROS-mediated p38MAPK/ERK signaling pathway	Attenuate renal fibrosis	[133]
BMSC-EVs	HK-2 cells	UUO	Inhibit RhoA/ROCK pathway	Attenuate renal fibrosis	[134]
BMSC-EVs	Pericytes; Fibroblasts; Macrophages	UUO	MiR-34c-5p inhibits the core fucosylation of multiple proteins	Ameliorate RIF	[135]
ADSCs-Exos	Podocyte	–	MiR-215-5p shuttles to podocyte, and inhibits the transcription of ZEB2	Improve podocyte dysfunction and DN symptoms	[136]
ADSCs-Exos	Podocyte	Spontaneous diabetes mice	Enhance the expression of miR-486, inhibit of Smad1/mTOR signaling pathway	Ameliorate DN symptom	[137]
hUC-MSC-EVs	HK-2 cells^*^	Diabetes and hyperuricemia mice	MiR-451a decreases α-SMA and increases e-cadherin expression by targeting 3′-UTR sites of P15 and P19	Decrease the morphologic and functional injury of kidney	[138]
BMSC-EVs	Renal tissue	Streptozotocin-induced diabetes mellitus rat	Enhance the expression of LC3, Beclin-1 and decrease the level of mTOR and fibrotic marker	Attenuate DN symptom	[139]
hBMSC-EVs	Glomerulus	NOD/SCID/IL2Rγ KO (NSG) mice	Downregulate Serpina1a, FAS ligand, CCL3, TIMP1, MMP3, collagen I and SNAI1	Ameliorate renal fibrosis and the expression of collagen I, attenuate DN symptom	[140]

*ADSC* adipose-derived mesenchymal stem cell, *BMSC* bone mesenchymal stem cell, *DN* diabetic nephropathy, *EVs* extracellular vesicles, Exos exosomes, *RIF* renal interstitial fibrosis, *UC-MSC* umbilical cord mesenchymal stem cell, *UUO* unilateral ureteral obstruction.

^*^HK-2, human proximal tubular epithelial cell line.

**Table 7:** Summary of studies on the role of extracellular vesicles in lung fibrosis.

EVs source	Target cells or tissues	Animal model	Molecular mechanism	Action effect	Ref
BMSC-Exos	Lung macrophage	Hyperoxia-induced BPD	Suppress M1 macrophage production and enhance M2 macrophage generation	Improve lung function, decrease fibrosis and pulmonary vascular remodeling, and ameliorate pulmonary hypertension.	[144]
hBMSC-Exos	Lung macrophage	Bleomycin-induced pulmonary fibrosis	Regulate total lung imbalance of macrophage phenotype	Prevent or reverse lung fibrosis	[145]
UC-MSC-Exos	PAEC and PASMC	Monocrotaline-induced rat model of PH	Regulate Wnt5a/BMP signaling pathway	Attenuate pulmonary vascular remodeling and lung fibrosis	[146]
UC-MSC-Exos	Lung tissue	BPD	Immunomodulatory glycoprotein TSG-6	Improve pulmonary inflammation, pulmonary simplification, pulmonary hypertension, and right ventricular hypertrophy	[147]
BMSC-EVs	IPF pulmonary tissue	IPF	MiR‐29b‐3p	Ameliorate IPF	[148]
BMSC-EVs	Lung fibroblast	PF	MiR-186 suppressed SOX4 and DKK1 expression, blocked fibroblast activation	Ameliorate IPF	[149]
hPMSC -EVs	Lung fibroblast	Whole thorax irradiation mouse model	MiR-214-3p downregulate ATM/P53/P21 signaling	Relieve radiation-induced lung inflammation and fibrosis	[150]
MenSCs-Exos	Recipient alveolar epithelial cells	BLM	MiRNA Let-7 suppresses ROS, mtDNA damage, and NLRP3 inflammasome activation	Remit pulmonary fibrosis	[151]
MSC-Exos	MLE-12 cells^*^	LPS-induced ALI	Transmit miR-23a-3p and miR-182-5p to inhibit NF-κB and hedgehog pathways	Reversed the LPS-induced lung injury and fibrosis	[152]

*ALI* acute lung injury, *BLM* bleomycin, *BMSC* bone mesenchymal stem cell, *BPD* bronchopulmonary dysplasia; *EVs* extracellular vesicles, *Exos* exosomes, *hPMSC* human placenta-derived mesenchymal stem cell, *IPF* idiopathic pulmonary fibrosis, *LPS* lipopolysaccharide, *PAEC* pulmonary artery endothelial cell, *PASMC* pulmonary vascular smooth muscle cell, *PF* pulmonary fibrosis, *PH* pulmonary hypertension, *MenSCs* menstrual blood-derived stem cell, *UC-MSC* umbilical cord mesenchymal stem cell.

*MLE-12, mouse lung epithelial cell line.

### Support tissue repair

#### Osteoarthritis

Osteoarthritis (OA) is the principal form of joint disease with unclear pathogenesis, presenting with pain and stiffness, and in some cases, disability [84]. Recently, MSC-EVs have been proven to have both regenerative and immunoregulatory benefits in OA (Table 2).

Several studies have reported that hBMSC-EVs play a significant role in the treatment of OA by inhibiting some pro-inflammatory pathways and factors, and enhancing the proliferation and migration of chondrocytes. Vonk et al. determined that MSC-EVs blocked NFκB signaling by inhibiting phosphorylation of IκBα, thereby down-regulating TNF-α-induced COX2 expression, and interleukins and collagenase activity. Additionally, MSC-EVs up-regulated the expression of SOX9 and WNT7A, and promoted the production of proteoglycan and type II collagen in in vitro studies [85]. Li et al. concluded that hBMSC-EVs promoted OA-chondrocyte (OA-CH) proliferation and migration and reduced apoptosis via downregulation of MMP13, ALPL, IL-1β-activated pro-inflammatory Erk1/2, PI3K/Akt, p38, TAK1, and NF-κB signaling pathways and increased gene expression of PRG4, BCL2, and ACAN (aggrecan) [86]. In addition, in OA-like chondrocytes, MSC-EVs induced the expression of type II collagen and aggrecan (chondrocyte markers), while inhibiting MMP-13 and ADAMTS5 (catabolic) and iNOS (inflammatory markers). In a CIOA model, treated mice also exhibited reduced cartilage and bone degeneration [87]. In an OA model, Ruiz showed that the effect of MSC-EVs was due to the presence of TGFBI mRNA and protein [88]. Analogously, in the same model, BMSC-EVs promoted the conversion of RAW264.7 from M1 to M2, reduced the expression of proinflammatory cytokines IL-1β, TNF-α, and IL-6, and enhanced the expression of IL-10, chondrogenic genes, collagen II and SOX9 [89]. Interestingly, Woo et al. revealed in their monosodium iodoacetate (MIA) rat and the surgical destabilization of the medial meniscus (DMM) mouse model that MSC-EVs could ameliorate cartilage degeneration by increasing type II collagen synthesis and decreasing MMP-1, MMP-3, MMP-13 and ADAMTS-5 expression in the presence of IL-1β [90].

Recent studies have also examined the effect of miRNAs in MSC-EVs. In synovial-derived MSC-EVs (SMSC-EVs), Tao et al. overexpressed miR-140-5p to block Wnt5a and Wnt5b to activate YAP via the Wnt signaling pathway and significantly reduce extracellular matrix (ECM) secretion [91]. Wang et al. found that exosomes derived from miR 155-5p–overexpressing SMSCs (SMSC-155-5p-Exos) promoted ECM secretion by targeting Runx2, which enhanced cartilage regeneration and ameliorated OA [92]. Likewise, SMSC-EVs highly expressed miR-31 and relieved OA via the KDM2A/E2F1/PTTG1 axis [93]. Of interest, hypoxia increased the expression of miR-216a-3p in HIF-1α-induced BMSC-EVs and promoted down-regulation of JAK2, promoting proliferation, migration, and reduced apoptosis of chondrocytes via inhibition of the JAK2/STAT3 signaling pathway [94]. A combination of these miRNAs and MSC-EVs may serve as a potential therapy for OA. In contrast, several studies have shown that miRNAs cause side effects in OA. Intra-articular injection of antagomir-miR-100-5p dramatically attenuated the infrapatellar fat pad (IPFP) MSC-EV (MSC^IPFP^-EVs)-mediated protective effect on articular cartilage in vivo [95]. MiR-29b-3p targets FoxO_3_ gene and enhances chondrocyte destruction. lncRNA H19 from umbilical cord MSC-EVs could competitively bind to miR-29b-3p to attenuate its inhibition of the target gene FoxO_3_ [96].

#### Spinal cord injury

Spinal cord injury (SCI) arises following damage to its structure and function by various pathogenic factors, with consequent spinal cord dysfunction including that of movement, sensation, and reflexes [97]. Due to the limited regenerative ability of nerve components, MSC-EVs have been recently viewed as a promising clinical treatment for SCI (Table 3).

A rat model of SCI has commonly been applied to evaluate treatment with MSC-EVs. They have been found to be able to regulate immunity and restore function through a variety of pathways. First, Huang et al. studied the administration of hBMSC-Exos in an animal model, and demonstrated that inhibition of apoptosis protein (Bax) and pro-inflammatory factors (TNFα and IL 1β), and promotion of anti-apoptotic protein (Bcl-2), anti-inflammatory protein (IL 10) and angiogenesis, could improve motor function [98]. Interestingly, the reduced pericyte migration mediated by BMSC-EVs correlated with inhibition of the NF-KB P65 signaling pathway with consequent weakening of the blood-spinal cord barrier (BSCB) [99]. In addition, Zhou et al. showed that treatment with BMSC-Exos suppressed the expression of caspase 1 and IL 1β by reducing pyroptosis, and enhanced neuronal regeneration to ameliorate motor ability in rats with spinal cord injury [100]. Han et al. found that TGF-β in BMSC-EVs enhanced the expression of Smad6, inhibited the excessive differentiation of neural stem cells (NSCs) into astrocytes, and promoted regeneration of neurons [101]. Consecutively, Nakazaki et al. proposed that BMSC-EVs should be administered over 3 days to up-regulate transforming growth factor -β (TGF-β), TGF-β receptor, and relative proteins of tight junction [102]. More intriguingly, Zhou et al. provided evidence that exosomes secreted by hPMSCs increased the activation of proliferating endogenous nerve stem/progenitor cells in vivo, while promoting NSC proliferation and upregulating MEK, ERK, and CREB phosphorylation levels in vitro, resulting in functional recovery [103].

MiRNAs have always been potent biological effectors of MSC-EVs, and without exception, they play a strong role in immune regulation and regeneration in spinal cord injury. Jia et al. confirmed that overexpression of miR-381 in MSC-EVs could promote SCI repair by up-regulating Ras homologous A (RhoA)/ RHO kinase activity and down-regulating BRD4 expression and DRG cell apoptosis by WNT5A [104]. Li et al. observed that miR-133 carried by MSC-Exos could directly target and down-regulate the expression of RhoA, and also promote expression of ERK1/2 STAT3 and CREB signaling pathway proteins related to neuronal survival and axon regeneration, thus rescuing neuron apoptosis and promoting axon regeneration [105]. Of interest, when miR-17-92, miR-26a, and miR-216a-5p were enriched in BMSC-Exos, they respectively induced activation of mTOR/PI3K/Akt, PTEN/ Akt /mTOR, and the TLR4/NF-κB/PI3K/ Akt signaling pathway cascade, with consequent promotion of axonal regeneration and nerve function repair after SCI [106–108]. In addition, miRNA-22 encapsulated in BMSC-EVs promotes neurogenesis and inflammation suppression by downregulating the expression of inflammatory cytokines and GSDMD, and blocking the pyroptosis of microglia after SCI [109]. Overexpression of miR-199a-3p/145-5p in exosomes secreted by human umbilical cord-derived MSCs has been shown to activate the NGF/TrkA signaling pathway affecting TrkA ubiquitination, and improve locomotor function in rats with SCI [110].

#### Skin injury

Skin injury is quite common. Skin regeneration is typically accompanied by four overlapping processes: inflammation, angiogenesis, new tissue formation, and remodeling [111–113] (Table 4).

There is recent evidence that human-derived MSC-Exos effectively benefit skin damage and accelerate wound healing by modulating related signaling pathways. Intriguingly, Zhou et al. adopted a combination therapy, applying hADSC-Exos both locally and intravenously to accelerate skin wound healing. Mechanistically, hADSC-Exos achieved this effect by down-regulating TNF-α, IL-6, CD14, CD19, CD68, and C-caspase 3, and up-regulating VEGF, CD31, Ki67, PCNA, filaggrin, loricrin and AQP3 [114]. Jiang et al. demonstrated that hBMSC-Exos suppressed TGF-β1, Smad2, Smad3, and Smad4 by targeting the TGF-β/Smad signaling pathway, but increased the expression of TGF-β3 and Smad7, thus improving scar formation and promoting wound healing [115]. Remarkably, fetal dermal mesenchymal stem cell-derived exosomes (FDMSC-Exos) have been shown to activate adult dermal fibroblast (ADFs) to promote cell proliferation, migration and secretion by targeting Jagged 1 ligand in the Notch signaling pathway, and ultimately accelerate wound healing [116].

Similar effects have also been observed for human-derived MSC-Exos carrying miRNAs. Of interest, He et al. showed that hBMMSCs and jaw bone marrow MSCs (JMMSCs) could induce macrophages toward M2 polarization and promote wound healing. The mechanism suggested that exosomes secreted by donors may regulate the polarization of macrophages by carrying miR-223 targeting Pknox1. Nonetheless, researchers cannot confirm whether other miRNAs or factors carried by these exosomes are involved in the induction of M2 polarization, and further studies are needed [117]. Likewise, Wu et al. utilized BMSC-Exos treated with 50 µg/mL Fe_3_O_4_ nanoparticles and 100 mT SMF to form a functional exosome (mag-BMSC-Exos). Notably, miR-21-5p was overexpressed in mag-BMSC-Exos and promoted angiogenesis in vivo and in vitro to accelerate skin wound healing by targeting SPRY2 to activate the PI3K/AKT and ERK1/2 signaling pathways [118]. Additionally, Cheng et al. found that hUCMSCs-EVs are highly enriched with miR-27b and promote the expression of JUNB and IRE1α by targeting the Itchy E3 ubiquitin-protein ligase (ITCH), thereby accelerating cutaneous wound healing [119]. In addition, hUMSC-Exos can be enriched with a set of microRNAs (miR-21, -23A, -125b, and -145) to attenuate excess myofibroblast formation and scarring via repression of the TGF-β2 /SMAD2 pathways [120]. Another study showed that hADSC-Exos derived miR-19b regulate the TGF-β pathway by targeting CCL1 [121]. Li et al. verified that hADSC-Exos down-regulated the expression of Col1, Col3, α-SMA, IL-17RA, and P-SMad2/P-SMad3, and up-regulated the level of SIP1 by suppressing multiplication and migration of hypertrophic scar-derived fibroblasts (HSFs). In addition, miR-192-5p was highly enriched in ADSC-EXO and reduced the level of pro-fibrosis protein, improved hypertrophic scar fibrosis, and accelerated wound healing via targeted inhibition of IL-17RA expression [122]. Alongside this, overexpression of miR-486-5P in hADSC-EVs enhanced the migration of human skin fibroblasts (HSFs) and the angiogenic activity of human microvascular endothelial cells (HMECs) by targeting Sp5 and motivating CCND2 expression, thereby promoting wound healing [123]. Interestingly, Gao et al. found that overexpression of Mir-135a in hAMSC-Exos significantly down-regulated LATS2, thereby increasing cell migration and promoting wound healing [124].

### Anti-fibrosis

#### Liver fibrosis

Liver fibrosis is a pathophysiological process and refers to the abnormal proliferation of intrahepatic connective tissue due to various pathogenic factors [125]. Recently, use of MSC-EVs has been considered a new therapeutic approach to repair liver fibrosis (Table 5). Rong et al. showed that human bone MSC-EVs inhibited expression of Wnt/β-catenin pathway components, α-SMA, and type I collagen, thereby preventing stellate cell activation and increasing hepatocyte regeneration. In vivo injection of hBMSC-Exos has been shown to effectively alleviate CCL4-induced liver fibrosis in rats and restore liver function [126]. Likewise, using a CCL4-induced liver fibrosis animal model, Ohara et al. proved that EVs from amnion-derived MSCs (AMSC-EVs) could significantly reduce the number of Kupffer cells (KCs), mRNA expression of inflammatory factors, activation of hepatic stellate cells (HSC), and the lipopolysaccharide (LPS)/toll-like receptor 4 (TLR4) signaling pathway, thereby reducing inflammation and fibrosis [127].

The anti-fibrotic effect of miRNAs in MSC-EVs has become a focus of research into CCL4-induced liver fibrosis in rats. MiRNA-181-5p overexpression in ADSC-EVs has been shown to down-regulate transcription 3 (STAT3) and Bcl-2 and activated autophagy in HST-T6 cells, alongside a significant decrease in collagen I, vimentin, a-SMA, and fibronectin in liver [128]. Similarly, high expression of miR-122 in ADSC-EVs modulated the expression of target genes such as insulin-like growth factor receptor 1 (IGF1R) cyclin G(CCNG1), and proline-4-hydroxylase A1(P4HA1), thereby more effectively blocking the proliferation of HSCs and collagen maturation [129]. Interestingly, Kim et al. reported that miR-486-5p was highly expressed in T-MSC-EVs that could target the hedgehog receptor, smoothened (Smo), and inhibit hedgehog signaling, thereby attenuate the activation of HSCs and liver fibrosis [130].

#### Kidney fibrosis

Renal fibrosis is a gradual pathophysiological process during which kidney function progresses from healthy to injured, then to damage with an ultimate loss of function [131]. Increasingly, MSC-EVs have been studied in the treatment of renal fibrosis using various models (Table 6).

Ji et al. determined that hUC-MSC-Exos repressed Yes-associated protein (YAP) through casein kinase 1δ (CK1δ) and E3 ubiquitin ligase β-TRCP in a rat model of unilateral ureteral obstruction (UUO), thus ameliorating renal fibrosis [132]. Similar effects in a UUO model were confirmed in Liu’s study. They revealed that hUC-MSC-Exos attenuated renal fibrosis by inhibiting the ROS-mediated p38MAPK/ERK signaling pathway [133]. Likewise, Shi et al. showed that milk fat globule–epidermal growth factor–factor 8 (MFG-E8) was included in BMSC-EVs, and ameliorated renal fibrosis by blocking the RhoA/ROCK pathway in a UUO model [134]. Of interest, in a UUO mouse model, BMSC-Exos loaded miR-34c-5p inhibited core fucosylation (CF) by cd81-EGFR complex, thereby improving renal interstitial fibrosis (RIF) [135]. Correspondingly, recent studies also suggest that exosomes from ADSCs ameliorate the development of DN via miRNAs. Jin et al. used miRNA-215-5p to inhibit ZEB2 and improved diabetic nephropathy (DN) symptoms. They also revealed that upregulated expression of miR-486 could suppress the Smad1/mTOR signaling pathway in podocytes [136, 137]. MV-miR-451a from hUMSCs repressed cell cycle inhibitor P15 and P19 expression by targeting their 3′-UTR sites, thereby decreasing α-SMA and increasing e-cadherin expression. This resulted in epithelial-mesenchymal transformation (EMT) reversal and improved DN symptoms [138]. In another study of amelioration of DN, BMSC-Exos significantly enhanced the expression of LC3 and Beclin-1, and decreased the level of mTOR and fibrotic markers in a streptozotocin-induced rat model of diabetes mellitus [139]. Interestingly, Grange et al. reported that renal fibrosis and the expression of collagen I were significantly ameliorated via multiple injections of HLSCs (human liver stem-like cells) and MSC-EVs in NOD/SCID/IL2Rγ KO (NSG) mice. Additionally, related genes (Serpina1a, FAS ligand, CCL3, TIMP1, MMP3, collagen I, and SNAI1) were significantly downregulated, thereby attenuating DN symptoms [140].

#### Lung fibrosis

Pulmonary fibrosis is a terminal change in lung disease characterized by fibroblast proliferation and accumulation of a large amount of extracellular matrix accompanied by inflammatory injury and destruction of tissue. Normal alveolar tissue is damaged and abnormal repair leads to structural abnormalities [141, 142]. The etiology in the vast majority of patients with pulmonary fibrosis is unknown [143]. Idiopathic pulmonary fibrosis (IPF) manifests mainly with pulmonary fibrotic lesions and is a serious interstitial lung disease that can lead to progressive loss of lung function. IPF has a higher mortality than most tumors and is considered a tumor-like disease [142]. Recently, MSC-EVs have become an effective treatment for pulmonary fibrosis (Table 7).

BMSC-Exos exert their therapeutic effect through immunomodulation. In a mouse model, BMSC-Exos have been shown to significantly ameliorate hyperoxia (HYRX)-induced bronchopulmonary dysplasia (BPD), alveolar fibrosis, and pulmonary vascular remodeling by suppressing M1 macrophage production and enhancing M2 macrophage generation [144]. Likewise, BMSC-Exos have been shown to significantly reverse fibrosis in a bleomycin-induced pulmonary fibrosis model by regulating total lung imbalance of MΦ phenotype [145]. In addition, the Wnt5a/BMP signaling pathway regulated by UC-MSC-Exos can enhance Wnt5a, Wnt11, BMPR2, BMP4, and BMP9 expression, and down-regulate that of β-catenin, Cyclin D1 and TGF-β1. In a monocrotaline (MCT)-induced rat model of pulmonary hypertension (PH), MSC-Exos were shown to significantly ameliorate pulmonary vascular remodeling and pulmonary fibrosis [146]. Of interest, Chaubey et al. showed that UC-MSC-Exos played a therapeutic role in improving pulmonary inflammation, pulmonary simplification, pulmonary hypertension, and right ventricular hypertrophy through immunomodulatory glycoprotein TSG-6 in a neonatal BPD mouse model [147].

Additionally, MSC-EVs can reverse lung injury and pulmonary fibrosis by expressing influential miRNAs. Wan et al. determined that high expression of miR-29b-3p by BMSC-EVs ameliorated IPF by FZD6 [148]. Zhou et al. found that miR-186 enriched by BMSC-EVs repressed the expression of SOX4 and Dickkopf-1 (Dkk1), thereby effectively inhibiting fibroblast development and attenuating IPF [149]. In addition, Lei’s study revealed that hPMSC -EVs could carry miR-214-3p and downregulate ATM/P53/P21 signaling, thus relieving radiation-induced lung inflammation and fibrosis [150]. In BLM-induced lung fibrosis and a mouse model of alveolar epithelial cell damage, exosomes secreted from MenSCs (MenSCs-Exos) have been shown to ameliorate pulmonary fibrosis by transferring miRNA Let-7 to suppress reactive oxygen species (ROS), mitochondrial DNA (mtDNA) damage, and activation of NLRP3 inflammasome [151]. Similarly, Xiao et al. used another LPS-induced Acute Lung Injury (ALI) mouse model and demonstrated that MSC-Exos repressed NF-κB and hedgehog pathways by transporting miR-23a-3p and miR-182-5p, thereby improving lung injury and fibrosis [152].

## Challenges and application of MSC-EVS as an advanced therapy

Although MSC-EV-based therapy holds great promise as a novel “cell-free” therapeutic product, there remain many challenges to overcome prior to their clinical application. At present, several limitations restrict the clinical translation of MSC-EVs including the discrepancies in the components of EVs from various sources and the lack of standard operation processes for largescale production, both of which largely depend on quality control of the sources of EVs. It is plausible to overcome these hurdles by introducing a strategy to control the quality of MSCs from the original source of EVs.

### The quality of MSC-derived EVs from different groups and batches is heterogeneous

MSCs are most commonly derived from bone marrow, fat, umbilical cord and other tissues, but maintaining consistent quality of MSCs and their EVs from different sources and across batches is difficult. This severely restricts the quality control and management of MSCs and their EVs as drugs, and increases the problem of drug resistance [153]. This results in limited reproducibility of functional measurements in vitro and in vivo [154].

In the angiogenesis study, BMSC-, ADSC-, and UCBMSC-derived EVs were compared and found to reduce myocardial apoptosis, facilitate angiogenesis, and improve cardiovascular function. Notably, EVs from ADSCs stimulated cardioprotection factors VEGF, bFGF, and HGF [155]. In addition, BMSC-derived EVs appeared to have a greater angiogenic potential than ADSC-derived EVs when compared in two independent ischemic model studies, with an approximately 4-fold increase in endothelial cell numbers compared with controls, and a 1.5-fold change in the latter [156, 157]. Nonetheless, another study showed that EVs from endometrial mesenchymal stem cells resulted in a greater level of angiogenesis than EVs from BMSCS or ADMSCs [158].

In studies of osteogenesis studies, in two separate rat skull defect studies, BMSC-EV treatment increased bone volume four-fold relative to the control group [159], while ADSC-EV increased bone volume by about 1.33 times [160]. In other studies, BMSC- and ADSC-derived EVs accelerated chondrocyte proliferation, migration, and osteogenic differentiation [161, 162].

Comparison of the immunomodulatory differences of MSC-derived EVs from different sources revealed that BMSC-EVs and ADSC-EVs could induce M2 polarization of macrophages in vivo and in vitro [163, 164]. Interestingly, in a separate experiment, Wang et al. showed that BMSC-EVs prompted a significant (3.2-fold) increase in the expression of CD206 of M2-polarization marker in an acute lung injury mouse model [163]. Nonetheless Liu et al. reported that the M2 polarization ability of ADSC-EVs increased only by a factor of 1.5 in a mouse model [165].

### The proliferation capacity of MSCs extracted from adult tissues was limited, and affected the largescale production of EVs

To develop MSC-EVs into commercially advanced therapeutic products (ATPs), quality assurance (QA) is required of the original material, including parental groups or cells used in the manufacture of MSCs. There remain many difficulties in mass production of EVs from adult tissues for clinical trials since proprietary MSCs have a limited number of passage times, age easily, and come at a high financial cost. In addition, their heterogenicity makes traditional cell culture inefficient in terms of time and cost.

### MSCs derived from pluripotent stem cells overcome the problems of mass production of MSC-EVs and quality heterogeneity

The original source MSCs requires good, consistent, and controllable quality, with a strong ability to proliferate and to secrete large numbers of EVs. To achieve this, we established an induction system of MSCs using pluripotent stem cells to overcome the problems of mass production of MSC-EVs and variation in quality. We successfully induced MSCs from pluripotent stem cells (PSC) [166–170]. Compared with MSCs extracted from traditional sources, our MSCs were derived from the same parent PSCs, consequently overcoming the problem of EV heterogeneity when MSCs from a variety of sources are used. Recently, GMP-grade MSCs derived from human PSCs (hPSC) have been used in clinical trials for refractory graft-versus-host disease (GVHD) [171]. The therapeutic potential of MSC-EVs has been shown in preclinical studies of both acute GVHD (aGVHD)[172–174] and chronic GVHD (cGVHD) [175] models. The preliminary benefits of hPMSC-EVs have been reported in a patient with cutaneous cGVHD. The stiffening and dryness of skin were improved significantly after intravenous injection of hPMSC-EVs [176]. Based on the preliminary efficacy and safety profiles, a phase 1 study has been launched to evaluate the safety and efficacy of BM-MSC-derived EVs in patients with acute or chronic rejection following abdominal solid organ transplantation (NCT05215288, Table 1). It is plausible that hPSC-MSC-derived EVs will promote the clinical translation of MSC-EVs owing to the quality control and largescale productive advantages of hPSC-MSCs compared with traditional MSC. hPSC-MSCs have more passages (more than 30 generations), strong amplification ability, can withstand senescence [166, 167, 170], and have strong secretion ability (including cytokines and exosomes) [168] compared with the traditional MSCs. Nonetheless, the passage times of traditional MSCs are generally less than 10 generations, and the proliferation and differentiation abilities of MSCs are reduced after numerous passages in culture, and affects the secretion of extracellular vesicles. Therefore, our hPSC-MSCs have great advantages for large-scale production and cost control of EVs. Mass production of MSCs and their EVs is now possible using bioreactors and microcarriers to maximize MSC growth and EV release per unit surface area. We evaluated mesenchymal stem cells from different sources and found that PSC-MSCs had the highest EV production. To optimize EV production, we acquired hPSC-MSCs in a scalable cell factory-based culture and were able to overcome the major obstacles during transformation of MSC-EVs into ATPs.

## Conclusions and future perspective

Extracellular vesicles derived from mesenchymal stem cells play a critical role in the development of immune regulation and regeneration. These EVs mimic the effects of stem cells and perform powerful functions by modulating immune pathways, promoting effector cell migration and proliferation, and reducing apoptosis. To date, 15 clinical trials have been registered in ClinicalTrial.gov, but none has been completed. Although EVs compared with MSC cell therapy incite a lower immune response and have a higher safety profile, there remain challenges to their clinical application [56]. In addition, the successful application of EVs depends on low cost for mass production, as well as improved separation efficiency and more accurate characterization methods. This review has discussed the therapeutic effects of EVs based on the function of MSCs or the introduction of specific molecules (such as miRNAs and lncRNAs). As work continues, researchers are actively developing engineered EVs that are more effective and capable of targeting, through loading of bioactive molecules and surface modification. Of interest, Feng et al. developed ε-polylysine-polyethylene-distearyl phosphatidylethanolamine (PPD) to modify MSC-EVs and invert their surface charge. As a result, the steric and electrostatic hindrance of cartilage matrix were alleviated, and the efficiency of MSC-EVs in the treatment of OA was improved [177]. These treatment strategies have achieved promising results at the initial stage and provide exciting new avenues for regenerative medicine therapy.

## Supplementary information

agreement about author list

## Data Availability

All relevant data are included in this manuscript.
