# Supplementary material for: Mesenchymal stem cell-derived extracellular vesicles for immunomodulation and regeneration: a next generation therapeutic tool?
Source: Cell Death Dis. 2022 Jul 4;13(7):580. doi: 10.1038/s41419-022-05034-x (PMC9252569; doi:10.1038/s41419-022-05034-x)
Supplement: Supplementary file 1 — agreement about author list [file 41419_2022_5034_MOESM1_ESM.docx]

1. **Author 1: Meng Kou**

**From:** koumengkara <[koumengkara@163.com](mailto:koumengkara@163.com)>
**Sent:** Tuesday, June 14, 2022 10:13
**To:** qzlian
**Subject:** Re: CDDIS-22-0452RR Initial Quality Check

Dear Professor Lian,

I agree with the changes to add these co-authors as below: Xiaoxian Zhang; Xiaoya Zhou; Aimin Xu; Hung-fat Tse.

Regards

Meng Kou

Cord Blood Bank Centre,

Guangzhou Women and Children's Medical Centre,

Guangzhou Medical University,

Guangzhou, China.

2. **Author 2: Li Huang**

**From:** Li Huang <[huanglth@163.com](mailto:huanglth@163.com)>
**Sent:** Tuesday, June 14, 2022 9:28
**To:** qzlian
**Subject:** Re:Fw: CDDIS-22-0452RR Initial Quality Check

Dear Prof. Lian,

Congratulations! Thanks for your information. I agree with the changes to add these co-authors as below: Xiaoxian Zhang; Xiaoya Zhou; Aimin Xu; Hung-fat Tse.

Regards

Li Huang

Guangzhou Women and Children's Medical Centre,

Guangzhou Medical University

3. **Author 3: Jinjuan Yang**

**From:** 杨金娟 <[jinjuanyang@163.com](mailto:jinjuanyang@163.com)>
**Sent:** Tuesday, June 14, 2022 10:08
**To:** qzlian
**Subject:** Re:Fw: CDDIS-22-0452RR Initial Quality Check

Confirmed, no objection

Jinjuan Yang

Guangzhou Women and Children's Medical Centre,

Guangzhou Medical University

4. **Author 4: Zhixin Chiang**

**From:** Chiang Zhi Xin <[chiangzhixin@gmail.com](mailto:chiangzhixin@gmail.com)>
**Sent:** Tuesday, June 14, 2022 18:10
**To:** qzlian
**Subject:** Re: Fw: CDDIS-22-0452RR Initial Quality Check

Dear Professor Lian,

I agree to the changes.

Thank you.

Regards,

Zhi Xin

Tunku Abdul Rahman University

5. **Author 5: Shaoxiang Chen**

**From:** 1271506997 <[1271506997@qq.com](mailto:1271506997@qq.com)>
**Sent:** Wednesday, June 15, 2022 19:57
**To:** qzlian
**Subject:** 回复：Fw: CDDIS-22-0452RR Initial Quality Check

Dear Prof. Lian,

Congratulations! Thanks for your information. I agree to add these co-authors as below: Xiaoxian Zhang; Xiaoya Zhou; Aimin Xu; Hung-fat Tse.

Regards

Shaoxiang Chen

Guangzhou Women and Children's Medical Centre,

Guangzhou Medical University,

Guangzhou, China.

6. **Author 6: Jie Liu**

**From:** liujie96 <[liujie96@hku.hk](mailto:liujie96@hku.hk)>
**Sent:** Saturday, June 18, 2022 18:00
**To:** qzlian;

**Subject:** Re: CDDIS-22-0452RR Initial Quality Check

Dear Professor Lian,

Yes, I confirm these changes, thank you!

Best regards,

Jie Liu

University of Hong Kong

7. **Author 7: Liyan Gou**

**From:** 郭立岩 <[liyanguos@163.com](mailto:liyanguos@163.com)>
**Sent:** Tuesday, June 14, 2022 11:37
**To:** qzlian
**Subject:** Re:Fw: CDDIS-22-0452RR Initial Quality Check

Dear Professor Lian，

I agree to these changes.

Sincerely

Liyan Guo

Prenatal Diagnostic Centre and Cord Blood Bank, Guangzhou Women and Children's Medical Center, Guangzhou, China

Postcode：510623

Tel: +86 17750593147

E-mail; [liyanguos@163.com](mailto:liyanguos@163.com)

8. **Author 8: Xiaoxian Zhang**

**From:** xx z <[zxx.merry@gmail.com](mailto:zxx.merry@gmail.com)>
**Sent:** Saturday, June 18, 2022 11:06
**To:** qzlian
**Subject:** Re: Fw: CDDIS-22-0452RR Initial Quality Check

Dear Prof. Lian,
Thank you for your information. I agree with the changes to add these co-authors as below: Xiaoxian Zhang; Xiaoya Zhou; Aimin Xu; Hung-fat Tse.

Regards
Xiaoxian Zhang
Cord Blood Bank Centre,
Guangzhou Women and Children's Medical Centre,
Guangzhou Medical University,
Guangzhou, China.

9. **Author 9: Xiaoya Zhou**

**From:** 周小雅 <[xiaoyawansi@163.com](mailto:xiaoyawansi@163.com)>
**Sent:** Tuesday, June 14, 2022 15:14
**To:** qzlian
**Subject:** Re:Fw: CDDIS-22-0452RR Initial Quality Check

Dear Dr. lian,

I confirm and agree to these changes，thank you very much!

Best regards,

Xiaoya Zhou

Guangzhou Women and Children's Medical Centre,
Guangzhou Medical University

10. **Author 10: Xiang Xu**

**From:** Xiang Xu <[xiangxu@tmmu.edu.cn](mailto:xiangxu@tmmu.edu.cn)>
**Sent:** Tuesday, June 14, 2022 11:07
**To:** qzlian
**Subject:** 回复: CDDIS-22-0452RR Initial Quality Check

Dear prof.lian,

Congratulations on the publication of the paper!

I completely agree these changes.

Thanks very much!

Best Wishes！

XiangXu

Daping Hospital,

Army Medical University

11. **Author 11: Xiaomei Yan**

**From:** xmyan <[xmyan@xmu.edu.cn](mailto:xmyan@xmu.edu.cn)>
**Sent:** Tuesday, June 14, 2022 10:00
**To:** qzlian
**Subject:** 回复: Fw: CDDIS-22-0452RR Initial Quality Check

Dear Prof. Lian,

Thanks for sharing this great news. Yes, I agree to these changes.

Best Regards,

Xiaomei

College of Chemistry and Chemical Engineering,

Xiamen University

12. **Author 12: Yan Wang**

**From:** 王焱 <[wy@medmail.com.cn](mailto:wy@medmail.com.cn)>
**Sent:** Wednesday, June 15, 2022 20:56
**To:** qzlian
**Subject:**

Prof. Lian, I aggree with the changes of more co-authors in our revised manuscript.

**Yan Wang**

School of Medicine,

Xiamen University

13. **Author 13: Jinqiu Zhang**

**From:** Jinqiu Bi <[zzzjinqiu@gmail.com](mailto:zzzjinqiu@gmail.com)>
**Sent:** Saturday, June 18, 2022 13:08
**To:** qzlian
**Subject:** Re: Fw: CDDIS-22-0452RR Initial Quality Check

Hi Dr Lian,

I agree with all the changes, thank you!

Jinqiu Zhang

Guangzhou Women and Children's Medical Centre,

Guangzhou MedicalUniversity

14. **Author 14: Aimin Xu**

**From:** amxu <[amxu@hku.hk](mailto:amxu@hku.hk)>
**Sent:** Wednesday, June 15, 2022 16:46
**To:** qzlian
**Subject:** Re: CDDIS-22-0452RR Initial Quality Check

Dear Dr. Lian, I agreed to the changes.

Best,

Aimin Xu

University of Hong Kong

15. **Author 15: Hung-fat Tse**

**From:** hftse<[hftse@hku.hk](mailto:hftse@hku.hk)>
**Sent:** Friday, June 17, 2022 19:53
**To:** qzlian
**Subject:** Re: 回复：Fw: CDDIS-22-0452RR Initial Quality Check

Agreed

**Hung-fat Tse**

University of Hong Kong
